# RNA-seq based transcriptomic analysis uncovers α-linolenic acid and jasmonic acid biosynthesis pathways respond to cold acclimation in *Camellia japonica*

**DOI:** 10.1038/srep36463

**Published:** 2016-11-07

**Authors:** Qingyuan Li, Sheng Lei, Kebing Du, Lizhi Li, Xufeng Pang, Zhanchang Wang, Ming Wei, Shao Fu, Limin Hu, Lin Xu

**Affiliations:** 1 Forestry and Fruit Tree Research Institute, Wuhan Academy of Agricultural Science and Technology, Wuhan 430075, China; 2 College of Horticulture and Forestry Sciences, Huazhong Agricultural University, Wuhan 430070, China; 3 National Key Laboratory of Crop Genetic Improvement, Wuhan 430070, China

## Abstract

Camellia is a well-known ornamental flower native to Southeast of Asia, including regions such as Japan, Korea and South China. However, most species in the genus *Camellia* are cold sensitive. To elucidate the cold stress responses in camellia plants, we carried out deep transcriptome sequencing of ‘Jiangxue’, a cold-tolerant cultivar of *Camellia japonica*, and approximately 1,006 million clean reads were generated using Illumina sequencing technology. The assembly of the clean reads produced 367,620 transcripts, including 207,592 unigenes. Overall, 28,038 differentially expressed genes were identified during cold acclimation. Detailed elucidation of responses of transcription factors, protein kinases and plant hormone signalling-related genes described the interplay of signal that allowed the plant to fine-tune cold stress responses. On the basis of global gene regulation of unsaturated fatty acid biosynthesis- and jasmonic acid biosynthesis-related genes, unsaturated fatty acid biosynthesis and jasmonic acid biosynthesis pathways were deduced to be involved in the low temperature responses in *C. japonica*. These results were supported by the determination of the fatty acid composition and jasmonic acid content. Our results provide insights into the genetic and molecular basis of the responses to cold acclimation in camellia plants.

Temperature is an important environmental factor that affects all living organisms, and cold stress severely alters plant growth, development, productivity and distribution^1^. Plants from temperate regions vary dramatically in their ability to survive freezing temperatures and can increase their freezing tolerance during exposure to chilling and non-freezing temperatures, which is known as cold acclimation^2^. Cold acclimation is a complex process and plants have developed intricate regulatory mechanisms to adapt to low temperatures. During this process, a series of protective mechanisms, including accumulation of cryoprotectant molecules, such as soluble sugars and sugar alcohols, synthesis of antifreeze proteins, and increases in the scavenging activity of reactive oxygen species (ROS), are induced^3^. Furthermore, alterations in the expression of many cold-related genes have been confirmed to regulate these aforementioned changes^2^.

The plasma membrane is the primary site of freezing injury. One of the key functions of cold acclimation is to stabilize membranes against freezing injury^2^. The lipid composition is a key factor in the stabilization of membranes and the enhancement of cold tolerance. The amount of unsaturated fatty acids in the plastid membranes of cold-resistant plants is greater than that of cold-sensitive plants^4^, suggesting that the level of unsaturated fatty acids is associated with the cold tolerance. The maintenance of polyunsaturated fatty acid levels in chloroplast lipids has been shown to contribute to survival in low temperatures and the normal formation of chloroplast membranes in plants under cold stress^5^. Fatty acid desaturases (FADs) are important enzymes involve in fatty acid desaturation in plants^5^. Both the Arabidopsis *fad5* mutant, which lacks an active chloroplast ω-9 FAD, and the *fad6* mutant, which lacks an active ω-6 FAD, have reduced levels of polyunsaturated fatty acids in the chloroplast galactolipids and reduced cold tolerance^6^. Trienoic fatty acids (TAs), such as hexadecatrienoic acid (16:3) and linolenic acid (18:3), the major polyunsaturated fatty acid species in plant membrane lipids, are important for ensuring the normal biogenesis and maintenance of chloroplasts during plants growth under low temperatures^7^. Moreover, the *Arabidopsis fad2* mutant defective in an oleate desaturase showed a decreased polyunsaturated fatty acid content, abnormal membrane rigidification and diacylglycerol kinase activation, supporting the hypothesis that plant cells can sense cold stress via its membrane rigidification^3^,^8^.

In addition to their key roles in signal perception and transmission, plant plasma membranes are themselves an important source of signalling molecules, many of which are derived from fatty acids^9^. Jasmonic acid (JA) and its derivatives are the best-studied fatty acid-derived signalling molecules, and the fatty acid substrate of JA biosynthesis is α-linolenic acid (18:3), which is released from the galactolipids of the chloroplast^10^. Jasmonates (JAs) are important regulators of the plant response to abiotic stress, such as cold stress, as well as biotic stress and development^11^. The application of exogenous methyl jasmonate (MeJA) in wheat seedlings enhanced cold tolerance, and increase both antioxidase activities and soluble protein content^12^. Moreover, MeJA treatment significantly increased endogenous JA accumulation and induced cold tolerance in banana fruit during cold storage^13^. Similarly, physiological studies showed that exogenous MeJA application also increased the cold tolerance in peach and pomegranate fruits during low temperature storage^14^,^15^. A recent study showed that cold stimulated the germination of dormant seeds and promoted a transient increase in JA content in wheat, indicating that JA may promote the release of dormancy via cold stratification^16^. Molecular studies also indicated that the JA signalling pathway plays an important role in the early cold response by modulating the CBF signalling pathway^17^,^18^. However, the molecular regulation mechanisms of JA in plant cold responses are still largely unknown.

Camellias, the general name of species and cultivars with ornamental values in the genus *Camellia*, family Theaceae, are widely distributed in Southeast Asia, including regions such as Japan, korea and South China^19^. Most camellia plants are cold sensitive^20^,^21^. However, a wild population of *Camellia japonica L*., named Naidong Shancha by the natives and distributed in Lao Mountain and the nearby islands in Shandong province of China, showed a high cold tolerance^22^ and can be used for cold-tolerant studies. Studies on the molecular and physiological mechanisms of cold tolerance in Naidong Shancha may provide a theoretical basis for the genetic improvement of cold tolerance in camellia plants. In this study, we performed a transcriptomic analysis of ‘Jiangxue’, a cold-resistant cultivar of Naidong Shancha, during cold acclimation using in-depth RNA sequencing (RNA-seq). Based on the analysis of complex regulatory networks and differentially expressed genes, we found that genes related to transcription factors (TFs), protein kinases, plant hormone signal transduction, unsaturated fatty acid biosynthesis and JA biosynthesis showed differences in expression. Detailed analyses of the gene expression, fatty acid composition, and JA contents revealed that the α-linolenic acid and JA biosynthesis pathways may play important roles in the cold responses in *C. japonica*. Our data provide insights into the genetic and molecular basis of the responses to cold stress in camellia plants.

## Results

### Cold tolerance evaluation in different *C. japonica* cultivars

**Figure 1:**
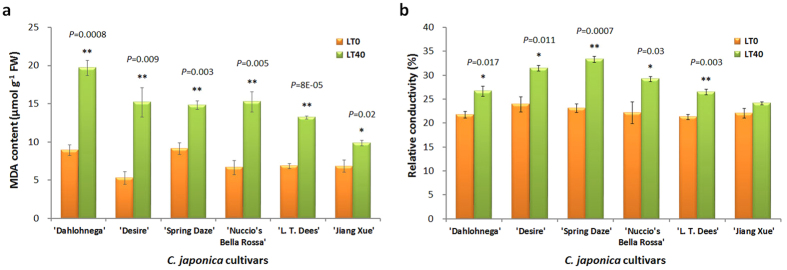
MDA and relative electrical conductivity change in different *C. japonica* cultivars that subjected to natural low temperature in 2013 winter. LT0 and LT40 represent plant that subjected to low temperature for 0 day and 40 days, respectively. The bars represent the standard error (n = 3). * and ** indicate a significant difference between LT0 and LT40 at P < 0.05 and P < 0.01 levels, respectively (two tailed T-test).

To evaluate the cold tolerance of different *C. japonica* cultivars, we subjected six *C. japonica* cultivars, ‘Dahlohnega’, ‘Desire’, ‘Spring Daze’, ‘Nuccio’s Bella Rossa’, ‘L. T. Dees’ and ‘Jiangxue’, to natural low temperatures from December 2013 to January 2014 (the detailed climatological data are shown in Supplementary Table 1). After the camellia plants underwent a period of 40 d under low temperatures, some cultivars, such as ‘Desire’ and ‘Nuccio’s Bella Rossa’ showed slightly cold injuries in young leaves and the edge of the petals, whereas no visible injuries were found in the cultivar ‘Jiangxue’. Cell damage in plants exposed to cold stress can be monitored by the relative electrical conductivity, or the malondialdehyde (MDA) content, which is a product of fatty acid degeneration and a marker for oxicative stress^23^,^24^. To study the cell damage in different *C. japonica* cultivars, the MDA content and relative electric conductivity were determined. In all *C. japonica* cultivars, the MDA content and relative electric conductivity increased after 40 d of low temperature compared with those in the plants at 0 d (Fig. 1). However, the increases in both MDA content and relative electric conductivity in the cultivar ‘Jiangxue’ were lower than those in the other cultivars (Fig. 1). These results suggested that the *C. japonica* cultivar ‘Jiangxue’ is more tolerant to cold stress than other cultivars. To elucidate how ‘Jiangxue’ addresses cold stress, we used ‘Jiangxue’ leaves during cold acclimation to obtain the transcriptomic response data.

### RNA sequencing and de novo transcriptome assembly

Plants in genus *Camellia* usually have large genome sizes, and thus far, the draft genome of *C. japonica* has not been sequenced and assembled. Therefore, to gain insight into the molecular mechanisms of cold tolerance in *C. japonica*, we used RNA sequencing in this study.

RNA was isolated from the leaves of ‘Jiangxue’ at 2, 8, 24, 72 and 168 h of 4 °C cold acclimation and control treatments. Three biological replicates were sampled. In total, 18 RNA samples were subjected to paired-end RNA sequencing. A total of 1,031 million raw reads were generated. After the low-quality reads were removed, and the adapter sequences were trimmed, 1,006 million (125.84 Gb) clean reads were obtained with an average of 55.9 million reads (6.99 Gb) for each sample (Supplementary Table 2).

The Trinity sequence assembler was used, and 367,620 transcript sequences and 207,592 unigenes were generated with transcript lengths ranging from 201 bp to 14,487 bp. The average length of an assembled transcript was 860 bases, and the N50 length was 1,415 bases (Supplementary Table 3). The length statistics of the assembled transcripts and unigenes are shown in Supplementary Fig. 1.

### Functional annotation of the assembled transcriptome

To predict and analyse the function of the assembled unigenes, we assessed the non-redundant sequences using a BLASTX search against the following databases: Nr (NCBI non-redundant protein sequences), Nt (NCBI non-redundant nucleotide sequences), Pfam (protein family database), Swiss-Prot (a manually annotated and reviewed protein sequence database), GO (Gene Ontology), COG (cluster of orthologous groups) and KEGG (Kyoto Encyclopaedia of Genes and Genomes). After the analysis, 65,150 (31.38%), 44,101 (21.24%), 47,011 (22.64%), 48,079 (23.16%) and 48,916 (23.56%) unigenes returned BLAST results and showed identity with sequences in the Nr, Nt, Pfam, SwissProt and GO databases, respectively. Overall, 84,910 (40.9%) unigenes were significantly matched to known genes in the public databases mentioned above (Supplementary Table 4).

**Figure 2:**
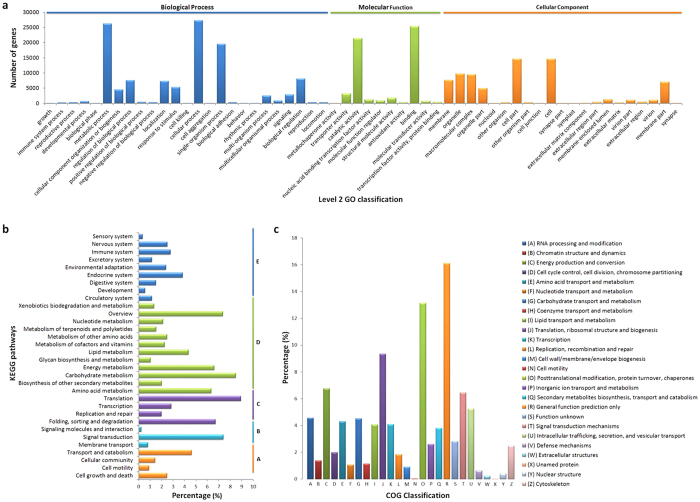
Functional annotation of assembled transcriptome. (**a**) GO classification of the annotated unigenes. The Y-axis represents the number of genes in a category. (**b**) KEGG classification of the annotated unigenes. The left Y-axis indicates the KEGG pathways, the right Y-axis indicates the sub-branches. A: cellular processes; B: environmental information processing; C: genetic information processing; D: metabolism; E: organismal systems. The X-axis indicates the percentage of unigenes that were assigned to a specific pathway. (**c**) COG classification of the putative proteins. The Y-axis indicates the percentage of unigenes in specific functional cluster.

To determine the potential functions of unigenes, we used GO assignments to classify the predicted *C. japonica* genes, and 48,916 unigenes were assigned to three major functional categories (Biological Process, Cellular Component and Molecular Function) and 56 subcategories (Fig. 2a). In terms of Biological Processes, ‘cellular processes’ and ‘metabolic processes’ were the top two GO terms, which indicated that the plants were undergoing rapid cell growth and were metabolically active. In the Molecular Function category, the unigenes were predominantly assigned to the ‘binding’ and ‘catalytic activities’ groups. In the binding subset, ‘organic cyclic compound binding’ and ‘heterocyclic compound binding’ were the most common groups. In the ‘catalytic activity’ subset, the two major groups were ‘transferase activity’ and ‘hydrolase activity’. In the Cellular Component category, the unigenes were frequently assigned to ‘cell’, ‘cell junction’, ‘macromolecular complex’ and ‘organelle’.

To identify the biological pathways in the annotated *C. japonica* sequences, we annotated the unigenes to the reference pathways in the KEGG using KeggArray software, and 23,476 unigenes were assigned to five specific pathways, including ‘Cellular Processes’, ‘Environmental Information Processing’, ‘Genetic Information Processing’, ‘Metabolism’, and ‘Organism Systems’ (Fig. 2b). Among these pathways, the ‘translation’ cluster represented the largest group, followed by ‘carbohydrate metabolism’ and ‘signal transduction’.

To classify the orthologous gene products, 23,514 unigenes were subdivided into 26 COG classifications (Fig. 2c). Among these classifications, the cluster of ‘general function prediction only’ represented the largest group, followed by ‘posttranslational modification, protein turnover, chaperones’ and ‘translation, ribosomal structure and biogenesis’. The two categories involving ‘cell motility’ and ‘unnamed protein’ represented the smallest COG classifications.

### Identification of genes involved in the cold response

The clean data from each sample were mapped onto the assembled transcriptome, and gene expression levels were estimated using RSEM (RNA-Seq by Expectation Maximization) for each sample. Genes with FPKM (fragments per kilobase of transcript per million fragments mapped) values equal to or larger than 0.3 were defined as expressed.

**Figure 3:**
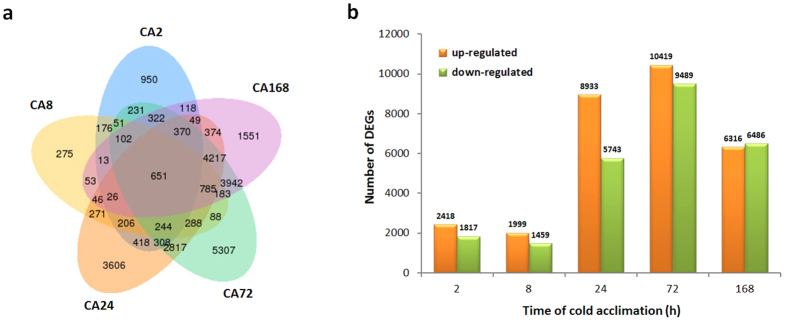
Venn diagram and histogram of differentially expressed genes during cold acclimation. (**a**) Venn diagram showing DEGs expressed at each of the five sampling time points during cold acclimation. CA2, CA8, CA24, CA72 and CA168 refer to 2, 8, 24, 72 and 168 h of cold acclimation. (**b**) The number of genes up- or down-regulated at different time points during cold acclimation.

To investigate genes involved in the cold response of *C. japonica* during cold acclimation, we identified the differentially expressed genes (DEGs) between cold-acclimated and non-acclimated samples using the DESeq R package, with an adjusted q value < 0.005. In total, 4235, 3458, 14,676, 19,908 and 12,802 DEGs were identified at 2, 8, 24, 72 and 168 h of cold acclimation, respectively. Among all DEGs, 651 DEGs were present at all five sampling time points, and 950, 275, 3606, 5307 and 1551 DEGs were specific for 2, 8, 24, 72 and 168 h of cold acclimation, respectively (Fig. 3a).

The number of genes up- or down-regulated at different sampling time points during cold acclimation is shown in Fig. 3b. The number of DEGs increased rapidly at 24 h of cold acclimation and reached to 19,908 at 72 h. In addition, there were more up-regulated genes than down-regulated genes during this period. The number of DEGs decreased at 168 h of cold acclimation, and the number of up-regulated genes was nearly equal to the number of down-regulated (Fig. 3b).

DEGs were divided into three groups based on their expression patterns by Genesis based on the K-means method (Supplementary Fig. 2). Type I genes, which included 13,663 genes, were positively modulated during cold acclimation and were divided into ten sub-clusters. Type II genes, which included 11,335 genes, were negatively affected during this process and were divided into nine sub-clusters. Type III represents genes showing complex expression patterns and was divided into four sub-clusters, containing 2710 genes.

**Figure 4:**
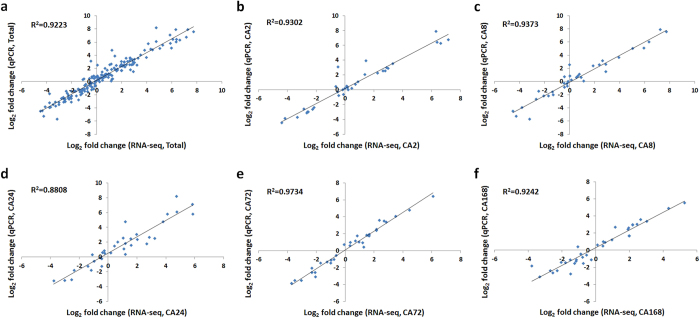
Correlation of gene expression ratios obtained by qPCR and RNA-seq. The qPCR log_2_ value of the expression ratio (cold-acclimated/non-acclimated) (y-axis) was plotted against the value from the RNA-seq (x-axis). All qPCR data were collected from three biological replicates and three technical replicates for each sample. CA2, CA8, CA24, CA72 and CA168 refer to 2, 8, 24, 72 and 168 h of cold acclimation.

To validate the expression profiles obtained by RNA-seq, we used qRT-PCR to confirm the expression of 35 genes that showed different levels during cold acclimation. The relative expression levels measured by qRT-PCR were converted to fold changes (cold-acclimated/non-acclimated) to enable a direct comparison with the RNA-seq data. The Pearson correlation coefficient was calculated by SPSS to assess the correlation between different platforms. Overall, the qRT-PCR measurements were highly correlated with the RNA-seq results (Fig. 4a, R^2^ = 0.9223; *p* = 7.87E-97). The correlations of gene expression levels estimated by qRT-PCR with those obtained by RNA-seq at different sampling time points ranged from 0.8808 to 0.9734 (Fig. 4b–f).

### Global gene regulation during cold acclimation

To survive in low temperatures, plants require an effective response to adapt to the low temperature. A rapid reaction may predominantly rely on a gene network involving TFs, protein kinases, plant hormone signal transduction pathways, unsaturated fatty acid biosynthesis, and JA biosynthesis pathways.

#### TFs

A total of 1455 DEGs encoding putative TFs were identified in *C. japonica* during cold acclimation. These TFs were classified into 53 different common families based on the classification of their *Arabidopsis* homologues (Supplementary Table 5). Among these families, the WRKY family was the largest group (294, 20%), followed by the NAC family (239, 16%), the bHLH family (96, 7%), the ethylene-responsive TF (ERF) family (72, 5%) and C2H2 (65, 4%) (Supplementary Fig. 3). In the WRKY and bHLH TF families, the number of up-regulated genes was nearly equal to that of the down-regulated genes. In the ERF and C2H2 families, the number of up-regulated genes was substantially greater than that of the down-regulated genes; while there were more down-regulated genes than up-regulated genes in the NAC family (Supplementary Fig. 4).

#### Protein kinases

Protein phosphorylation plays a pivotal role in plant responses to cold stress^25^. In our study, 74 MAP kinase kinase kinases (MPKKK), 5 MAP kinase kinases (MPKK), 11 MAP kinases (MPK), 17 calcium dependent protein kinases (CPK) and 31 CBL-interacting protein kinases (CIPK) were identified in *C. japonica* during cold acclimation based on MPK, MPKK, MPKKK, CPK and CIPK gene family information in Arabidopsis^26^,^27^ using the BLASTP programme (Supplementary Table. 6). In the MPKKK family, the number of up-regulated genes was slightly more than that of the down-regulated genes. Interestingly, most of DEGs in the CIPK family, the CPK family, the MPK family and the MPKK family were induced by low temperature in *C. japonica* (Supplementary Fig. 5). These results indicated that protein phosphorylation may be induced by low temperature during cold acclimation in *C. japonica*.

#### Plant hormone signal transduction pathway

Groups of genes involved in different plant hormone signalling pathways, including auxin, gibberellic acid (GA), ethylene (ET), abscisic acid (ABA), JA, cytokinin (CK) and brassinosteroid (BR) pathways, were identified in *C. japonica* during cold acclimation. The detailed gene expression patterns and gene descriptions are shown in Supplementary Table 7. Among these groups, the GA, ET, ABA and JA signalling pathways were predominantly induced (Supplementary Fig. 6a), while the auxin, BR and CK signalling pathways were noticeably inhibited (Supplementary Fig. 6b). In the ABA signalling pathway, nine ABA receptor genes, six ABA-responsive element binding factor (ABF) genes, six serine/threonine-protein kinase SRK2 (snrk2) genes and three protein phosphatase 2C (PP2C) genes were differentially expressed during cold acclimation. Five ET receptor (ERT) genes and six ERF genes were up-regulated in the ET signaling pathway. Notably, most DEGs in JA signalling pathway, including four jasmonate-ZIM domain (JAZ) genes, four MYC-related genes, two jasmonate associated MYC-like (JAM) genes and one jasmonate resistant (JAR) gene, were up-regulated during cold acclimation. The above results suggested that plant hormones may play roles in the response to cold stress in *C. japonica*.

### Unsaturated fatty acid biosynthesis pathway

**Figure 5:**
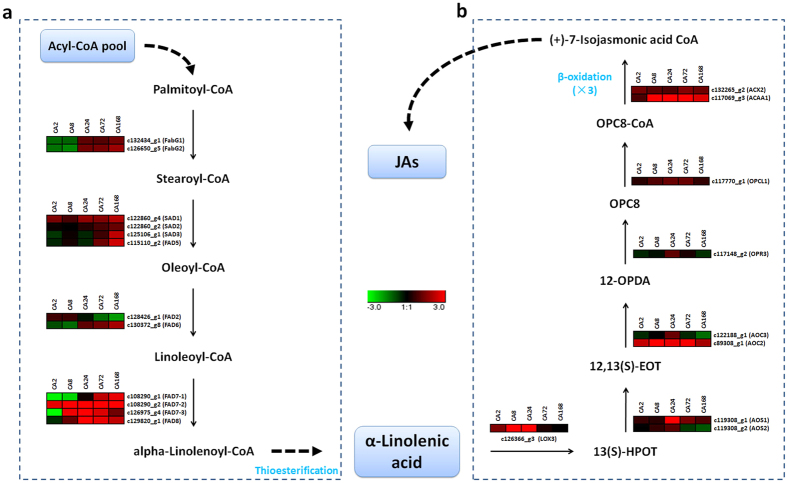
Overview of putative α-linolenic acid and JA biosynthesis pathways involved in *C. japonica* and expression profiles of genes involved in this pathway. The schematics of α-linolenic acid biosynthesis pathway (**a**) and JA biosynthesis pathway (**b**) were shown in brief. 13(S)-HPOT: (9Z,11E,15Z)-(13S)-13-Hydroperoxyoctadeca-9,11,15-trienoic acid; 12,13(S)-EOT: (9Z,15Z)-(13S)-12,13-Epoxyoctadeca-9,11,15-trienoic acid; 12-OPDA: (15Z)-12-Oxophyto-10,15-dienoic acid; OPC8: 8-[(1R,2R)-3-Oxo-2-{(Z)-pent-2-enyl}cyclopentyl]octanoate.

Unsaturated fatty acids are important in plasma membrane formation and stress tolerance. In this study, several octadeca-carbon (C18) unsaturated fatty acid biosynthesis genes were differentially expressed during cold acclimation as determined by RNA-seq analysis and are shown in a heat map (Fig. 5a). For example, two 3-oxoacyl-[acyl-carrier protein] reductase genes, *c108290*_ *g1 (FabG1*) and *c108290*_ *g2 (FabG2*), four stearoyl-CoA (Delta-9) desaturase genes, *c115110*_ *g2 (SAD1*), *c122860*_*G2 (SAD2*), *c122860*_ *g4 (SAD3*) and *c125106*_ *g1 (FAD5*), two omega-6 FAD genes, *c126650*_* g5 (FAD2*) and *c126975*_ *g4 (FAD6*) and four omega-3 FAD genes, *c128426*_ *g1 (FAD7-1*), *c129820*_ *g1 (FAD7-2*), *c130372*_ *g8 (FAD7-3*), *c132434*_ *g1 (FAD8*), were up-regulated during cold acclimation (Fig. 5a).

**Figure 6:**
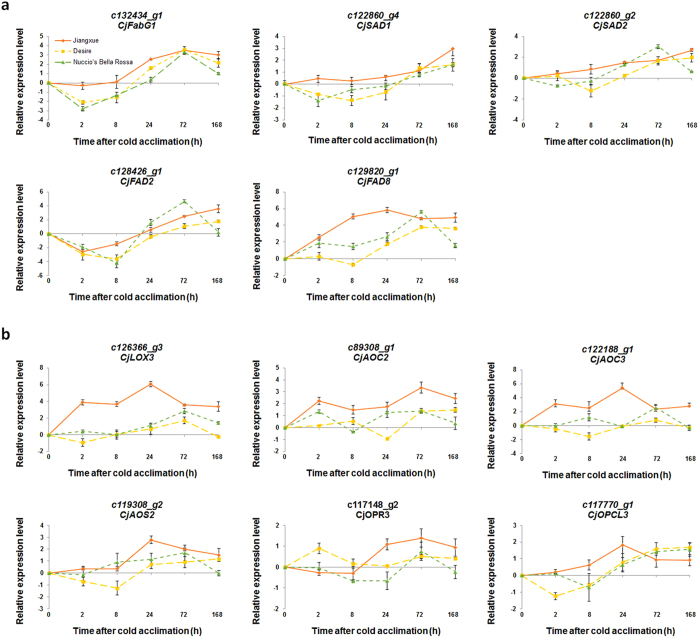
Detailed expression profiles of genes involved in α-linolenic acid and JA biosynthesis pathways. The relative expression level was obtained by qRT-PCR for data verification. Data in (**a**,**b**) are means ± SE from three biological independent experiments.

To confirm the results of the transcriptomic analysis, we verified the expression of several C18 unsaturated fatty acid biosynthesis genes using qRT-PCR. The expressions of *CjFabG1, CjSAD1, CjSAD2* and *CjFAD8* in ‘Jiangxue’ were induced during cold acclimation, especially after 24 h, while the *CjFAD2* transcripts were transiently inhibited in the early period (2 and 8 h) of cold acclimation and were then induced after 24 h (Fig. 6a). In addition, the expression of these genes in two other *C. japonica* cultivars ‘Desire’ and ‘Nuccio’s Bella Rossa’, which were more sensitive to cold stress compared with ‘Jiangxue’, were also determined. The expression of *CjFabG1, CjSAD1, CjSAD2* and *CjFAD2* were slightly inhibited at the early period (2 and 8 h) and then induced after 24 h, while the *CjFAD8* transcripts were almost continuous induced. However, the induction degrees of these genes by low temperature in ‘Desire’ and ‘Nuccio’s Bella Rossa’ were lower than that of in ‘Jiangxue’ (Fig. 6a). These results indicated that the unsaturated fatty acid biosynthesis pathway was activated by cold acclimation in *C. japonica*.

#### JA biosynthesis related-genes

JA and derivative compounds, collectively referred to as JAs, are key signalling molecules in plant stress responses and development^28^. In this study, several genes involved in JA biosynthesis, including one lipoxygenase gene, *c126366*_ *g3 (LOX3*), two hydroperoxide dehydratase genes, *c119308*_ *g1 (AOS1*) and *c119308*_ *g2 (AOS2*), two allene oxide cyclase genes, *c122188*_ *g1 (AOC3*) and *c89308*_ *g1 (AOC2*), one 12-oxophytodienoic acid reductase gene, *c117148*_ *g2 (OPR3*), one OPC-8:0 CoA ligase gene, *c117770*_ *g1 (OPCL1*), and two β-oxidase genes, *c132265*_ *g2 (ACX2*) and *c117069*_ *g3 (ACAA1*), were identified during cold acclimation. Notably, almost all these genes were up-regulated during cold acclimation in our expression profiling (Fig. 5b).

To further validate the above expression data, we verified the transcript levels of six JA biosynthesis genes using qRT-PCR. As shown in Fig. 6b, the expression of *CjLOX3, CjAOC2, CjAOC3, CjAOS1, CjOPR3* and *CjOPCL1* was strongly up-regulated during cold acclimation in ‘Jiangxue’ leaves, consistent with the results of expression profiling. However, the expression of these genes in ‘Desire’ and ‘Nuccio’s Bella Rossa’ during cold acclimation just slightly up-regulated (Fig. 6b). Together with the activated JA signalling transduction pathway (Supplementary Fig. 6a), the whole JA signalling pathway may play key roles in cold acclimation in *C. japonica*.

### Both α-linolenic acid and JA content were enhanced in *C. japonica* leaves during cold acclimation

**Figure 7:**
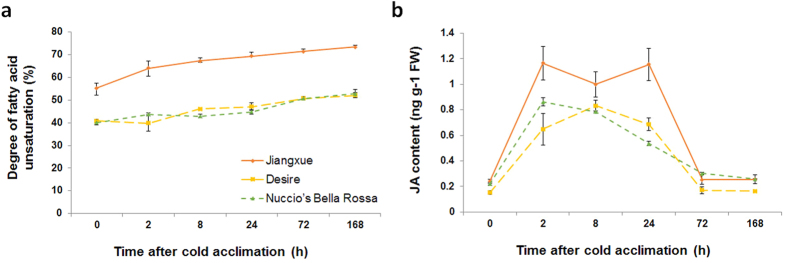
Degree of fatty acid unsaturation and endogenous JA level in *C. japonica* leaves during cold acclimation. The degree of fatty acid unsaturation (**a**) were presented as the percentage of unsaturated fatty acid in the total fatty acid. (**b**) JA contents in *C. japonica* during cold acclimation. Data in (**a**,**b**) are means ± SE from six biologically independent experiments.

To confirm that the unsaturated fatty acid biosynthesis pathway is important for the cold response in *C. japonica*, we measured the leaf fatty acid compositions in different *C. japonica* cultivars during cold acclimation using gas chromatography. As shown in Table 1, in ‘Jiangxue’, the content of palmitic acid (16:0), stearic acid (18:0) and oleic acid (18:1) gradually decreased during cold acclimation, while the content of α-linolenic acid (18:3) increased significantly during the same process, consistent with the up-regulated expression of unsaturated fatty acid biosynthesis genes. Similar tendencies were found in ‘Desire’ and ‘Nuccio’s Bella Rossa’, but the changes were not as significantly as that of in ‘Jiangxue’. Moreover, the degree of fatty acid unsaturation in all *C. japonica* cultivars gradually increased during cold acclimation, while both the degree of fatty acid unsaturation and its increment in ‘Jiangxue’ were remarkable higher than that of ‘Desire’ and ‘Nuccio’s Bella Rossa’ (Fig. 7a), which was consistent with the results from the gene expression analysis and the determination of fatty acid composition. These results indicated that the unsaturated fatty acid biosynthesis pathway, particularly the biosynthesis of α-linolenic acid (18:3), was involved in the cold response and was activated by cold acclimation to enhance cold tolerance in *C. japonica*s.

c (18:3) is the major substrate of JA biosynthesis^10^, and JA-related pathways, including the JA biosynthesis and JA signal transduction pathways, were activated during cold acclimation in this study. Therefore, we determined whether JA content was also increased during the cold acclimation process. The free JA levels in different *C. japonica* cultivars during cold acclimation were measured. In ‘Jiangxue’, the free JA content increased sharply after 2 h of 4 °C acclimation and was maintained at a high level from 2 h to 24 h compared with non-acclimated leaves. Then, at 72 h cold acclimation, the free JA contents decreased to the same level as that of the non-acclimated samples (Fig. 7b). Similar changes in JA content were found in ‘Desire’ and ‘Nuccio’s Bella Rossa’, while the increments of JA content in ‘Desire’ and ‘Nuccio’s Bella Rossa’ were lower than that of in ‘Jiangxue’. These results suggested that the activation of JA biosynthesis pathway at the early stage of cold acclimation may contribute to the cold tolerant in *C. japonica*.

## Discussion

Cold tolerance is an important trait in plants because it limits the geographical distribution of wild species and the growth performance and yield of crop plants. Unfortunately, most species in the genus *Camellia* are difficult to overwinter in high altitude cold regions because of their weak freezing tolerance^21^. Exploring the genetic resources and understanding the molecular and physiological mechanisms of chilling-resistant *Camellia* are needed to develop an effective strategy for genetic breeding of cold tolerance in camellia plants. In this study, we performed an in-depth transcriptomic analysis of the responses to low temperature in *C. japonica*. Our results revealed that complex gene networks, especially the unsaturated fatty acid and JA biosynthesis pathways, are involved in the cold acclimation process in *C. japonica*, further expanding our knowledge of how camellia plants increase cold-resistance after cold acclimation.

With the application of next-generation sequencing technology, plant genomic studies have been rapidly performed in recent years^29^. However, for species without a sequenced genome, such as the non-model plant species *C. japonica*, transcriptome sequencing is a rapid approach to study the regulatory pathways and molecular mechanisms of various complex physiological processes. Although several transcriptomic analysis in tea plants (*Camellia sinensis*) have been reported^23^,^30^, transcriptomic studies in *C. japonica* are still rare. In this study, we generated approximately 126 Gb of paired-end reads and obtained a reference transcriptome consisting of 367,620 contigs with a mean size of 860 bp and a N50 of 1415 bp (Supplementary Table 2 and 3), values much higher than those of the previously determined leaf reference transcriptome of other plants in the genus *Camellia*^23^,^30^. This large-scale and high-quality leaf-specific transcriptome data not only provided useful reference data but can also be used to elucidate cold stress response pathways and conduct subsequent functional genomics studies in *C. japonica* and other plants of the genus *Camellia*. To our knowledge, this is the first transcriptomic analysis of *C. japonica*. Moreover, a total of 84,910 of the 207, 592 unigenes from our unigene library were annotated to public databases (NR, NT, SwissProt, PFAM, GO and KOG) for comprehensive analysis (Supplementary Table 4). Compared with the previous studies in plants of the genus *Camellia*, which reported 43,201 annotated unigenes from 179,753 unigenes^23^ and 68,890 unigenes from 146,342 unigenes^30^, the present study obtained more complete annotation information.

TFs always act as master switches by controlling the expression of a series of genes to regulate different plant developmental processes or responses to biotic or abiotic stresses^31^. The WRKY family is one of the best-characterized classes of plant TFs, and many WRKY TFs have been shown to be involved in the cold response^32^. In this study, the WRKY family was the largest group involved in the cold acclimation process (Supplementary Figs 2 and 3; Supplementary Table 5), which indicated that increasing the tolerance to cold stress in *C. japonica* might require the complex mechanisms of signalling and transcriptional reprogramming controlled by WRKY proteins. The ERF family TFs are generally considered to be mediators of ethylene-related responses^33^. Genome-wide expression analyses of the AP2/ERF family genes indicated that many ERF family genes are also induced by low or high temperature^34^. In the present study, 72 DEGs encoding ERF family proteins were identified during cold acclimation and most of these genes were up-regulated (Supplementary Fig. 3; Supplementary Table 5), indicating that the induction of ERF family genes was required during the cold acclimation process in *C. japonica*.

MPK cascades convey stress signals from receptors to specific effectors to regulate gene expression, cell activities, and protein functions in various developmental and adaptive processes^35^,^36^. In our study, 75 MPKKK, 11 MPK and 5 MPKK genes were found to be involved in signal transduction upon low temperature stress (Supplementary Fig. 4; Supplementary Table 6), indicating that MAPK cascades play an important role in the cold acclimation process in *C. japonica*. CPK and CIPK, two protein kinase families known as Ca^2+^ sensors, have been shown to act as positive regulators of the plant cold response^27^,^37^,^38^. In our study, 31 CIPK and 17 CPK genes were differentially expressed during cold acclimation, and most of these genes were up-regulated (Supplementary Fig. 4; Supplementary Table 6). These results suggest that CPKs and CIPKs promote cold tolerance in *C. japonica*.

Phytohormones, such as ABA, JA, ET, SA, auxin, GA, CK, and BR, appeared to play critical roles in the complex signalling cascades and were essential for the plant adapation to biotic stresses by mediating a wide range of adaptive responses^39^. The ABA level increased in response to low temperatures in *Arabidopsis*^40^, and transcriptome analysis revealed that approximately 10% of ABA-responsive genes are also responsive to cold stress^41^. In the ABA signalling pathway, the ABA receptor, PP2C and SnRK2 form a signalling complex referred to as the ‘ABA signalosome’, which can turn on ABA signals by phosphorylation of downstream factors, such as the AREB/ABF TFs or membrane proteins involving ion channels^42^. As shown in this study, 24 genes in the ABA signalling pathway, including ABA receptor genes, SNRK2 genes, PP2C genes and ABF genes (Supplementary Fig. 5a; Supplementary Table 7), were differentially expressed during cold acclimation. These results thus indicate that the ABA-dependent cold signalling pathway may play important roles in *C. japonica*. JA and its derivatives are essential signalling molecules in plant developmental regulation and environmental stress responses^10^. A previous study showed that MaMYC2s, a key regulator of JA signalling, is involved in MeJA-induced chilling tolerance in banana fruit by interacting and functionally coordinating with MaICE1^13^. In the present study, four MYC genes, including two MYC2 genes and two MYC4 genes, were significantly induced during the cold acclimation process (Supplementary Fig. 5a; Supplementary Table 7), which indicates that the same regulatory pathways may exist in *C. japonica*. Furthermore, most DEGs in the JA signalling pathway were up-regulated in our study, indicating that JA may play a positive role in *C. japonica* adaptation to low temperatures.

Thus far, no specific receptor has been identified in plants in response to cold stress. However, the plasma membrane itself could be the primary sensor of temperature fluctuations^36^,^43^. Studies of the *Arabidopsis fad2* mutant have shown that low temperatures can be sensed by plant cells via membrane rigidification^3^,^8^. Other FAD genes were shown to be important for the membrane stabilization of plants in low temperature environments by maintaining high levels of polyunsaturated fatty acids^6^,^44^,^45^. In the present study, the expression of genes involved in several steps of the C18 unsaturated fatty acid biosynthesis pathway was up-regulated during cold acclimation (Figs 5a and 6a); as a result, α-linolenic acid (18:3), the end-product of this pathway, accumulated (Table 1). These results thus indicated that the C18 unsaturated fatty acid biosynthesis pathway was globally actived during cold acclimation in *C. japonica*. Moreover, a previous study showed that overexpression of the ω-3 FAD genes *BnFAD3* and *StFAD7*, which catalyse the conversion of linoleic acid (18:2) to linolenic acid (18:3) in tomato, increased the linolenic acid (18:3) level, while the level of linoleic acid (18:2) declined^46^. Consistent with the results from that study, our results showed that the precursors of linoleic acid (18:2) and α-linolenic acid (18:3), including palmitic acid (16:0), stearic acid (18:0) and oleic acid (18:1), were decreased in *C. japonica* leaves during cold acclimation (Table 1), while the degree of fatty acid unsaturation increased (Fig. 7a). Cold stress can change the membrane fatty acid compositions in many plants^5^,^24^. Therefore, the regulation of C18 fatty acid desaturation likely plays an important role in membrane stabilization under cold stress in *C. japonica*.

The substrate of JA biosynthesis is α-linolenic acid (18:3)^10^,^11^. A study of tomato showed that loss of function of a chloroplast FAD gene, *LeFAD7*, reduced the α-linolenic acid (18:3) content and impaired JA accumulation in defence responses^47^, suggesting that α-linolenic acid (18:3) in the chloroplast pools is required for stress-stimulated JA synthesis. Free α-linolenic acid (18:3) is oxygenated by lipoxygenase enzymes (LOX) and then converted to 12-oxo-phytodienoic acid (OPDA) through the combined action of allene oxide synthase (AOS) and allene oxide cyclase (AOC). OPDA is subsequently converted to JA by reduction and three cycles of β-oxidation^10^,^48^. In our study, the expression of nine JA biosynthesis genes, *LOX3, AOS1, AOS2, AOC2, AOC3, OPR3, OPCL1, ACX2, and ACAA1*, were up-regulated during cold acclimation (Figs 5b and 6b). In addition, our data further confirmed that the free JA content was increased at the early stage of cold acclimation in *C. japonica* leaves (Fig. 7b). Previous studies have shown that JA and its derivatives are important regulators of the plant responses to abiotic stresses^10^. The induced expression of JA biosynthesis genes and the increased free JA content in our study indicated that JA and its signalling pathway may play critical roles in the early stage of cold acclimation in *C. japonica*. Interestingly, a previous study showed that the ratio of unsaturated/saturated fatty acid in MeJA-treated loquat fruit was significantly higher than that in control fruit, along with reduced injury symptoms in MeJA-treated fruits^49^, indicating that JAs may also influence unsaturated fatty acid biosynthesis. In our study, both α-linolenic acid (18:3) and JA biosynthesis were enhanced during the cold acclimation process. However, whether α-linolenic acid (18:3) or JA acts as the upstream regulatory factor during the cold acclimation process in *C. japonica* will need to be determine in future studies.

## Methods

### Plant materials, growth conditions, and low temperature treatment

The *C. japonica* cultivars ‘Dahlohnega’, ‘Desire’, ‘Spring Daze’, ‘Nuccio’s Bella Rossa’, ‘L. T. Dees’ and ‘Jiangxue’ were planted in a green-house in Forestry and Fruit Tree Research Institute, Wuhan Academy of Agricultural Science and Technology, China. Camellia plants were moved to a nursery field and subject to natural low temperature at December 1, 2013. After the camellia plants underwent a period of 40 d under low temperatures, intact mature leaves were selected for MDA and relative electric conductivity determination.

For RNA-seq, *C. japonica* cultivar ‘Jiangxue’ were grown in growth chambers under long-day conditions (16 h light/8 h dark) under white fluorescent light at 25 °C during the day and 22 °C at night. Plants were then divided into two groups, the first group served as the control sample (CK), while the other was moved and cultured at 4 °C as the cold acclimation sample. Leaves of individual plants were collected for each treatment at each sampling time point.

### Determination of MDA and relative electric conductivity

The MDA content analysis was performed using the thiobarbituric acid method. First, 0.5 g fresh leaf samples were homogenized in 5 ml extraction buffer (10% trichloroacetic acid, w/v). After centrifugation at 10,000× g for 15 min, a 2 ml aliquot of the supernatant was heated with 2.0 ml of 0.5% (w/v) thiobarbituric acid at 100 °C for 30 min. Absorbance of the supernatants was measured at 450, 532, and 600 nm. The MDA content was calculated as described by Draper *et al*.^50^.

The relative electric conductivity was determined following the methods as described by Wang *et al*.^23^. Briefly, leaves were washed with deionized water. Then, 0.5 g midvein-excluded leaf samples were placed in closed vials containing 20 ml of deionized water and incubated at 25 °C on a rotary shaker for 24 h. The electrical conductivity of the solution (L1) was determined. Samples were then heated to 100 °C for 20 min and the final electrical conductivity (L2) was determined after equilibration at 25 °C. The relative electrical conductivity was defined as follows: relative electrical conductivity (%) = (L1/L2 × 100%).

### Sampling for RNA-seq and RNA preparation

Leaves of *C. japonica* cultivar ‘Jiangxue’ at 2, 8, 24, 72 and 168 h of cold acclimation and mixed samples of non-acclimated leaves at 2, 8, 24, 72 and 168 h (CK) were sampled for RNA isolation. Three independent biological replicates for each sampling point were sampled. Total RNA was extracted using a Plant Total RNA Extraction Kit (BioTeke, China) following the manufacturer’s instructions and then treated with RNase-free DNase I (Thermo Scientific, USA) to remove genomic DNA contamination.

### Library preparation and RNA sequencing

RNA samples were sent to Beijing Novogene Bioinformatics Technology Co., Ltd., where the libraries were produced and sequenced. Briefly, the RNA-seq libraries were generated using the NEBNext^®^ Ultra™ RNA Library Prep Kit for Illumina^®^ (NEB, USA) following manufacturer’s recommendations. mRNA was purified from total RNA using poly-T oligo-linked magnetic beads. Then, purification fragmentation buffer was added to cleave the mRNA molecules into short fragments. First-strand cDNA was synthesized using random hexamer primers and M-MuLV Reverse Transcriptase (RNase H-). Second-strand cDNA synthesis was subsequently performed using DNA Polymerase I and RNase H. NEBNext Adaptors with hairpin loop structures were ligated for hybridization. To select cDNA fragments of preferentially 150–200 bp in length, we purified the library fragments using the AMPure XP system (Beckman Coulter, Beverly, USA). Then, 3 μl USER Enzyme (NEB, USA) was used with size-selected, adaptor-ligated cDNA at 37 °C for 15 min followed by 5 min at 95 °C prior to PCR. Then, PCR was performed with Phusion High-Fidelity DNA polymerase, universal PCR primers and index (X) primer. Finally, the PCR products were purified (AMPure XP system), and library quality was assessed on an Agilent Bioanalyzer 2100 system. The resulting cDNA library was sequenced using an Illumina HiSeq platform. The original data set was deposited in the NCBI Sequence Read Archive (accession no. SRP076436).

### *De novo* transcript assembly and functional annotation of unigenes

Raw reads generated from sequencing were firstly processed through in-house Perl scripts. In this step, clean reads were obtained by removing reads containing adapter sequences, reads containing poly-N sequences and low quality reads from the raw data. First, bases with Phred quality scores lower than 20 were trimmed from the 3′ end of the sequence until reaching a base with a higher quality score (≥20). If the read length was shorter than 50 bp, it was discarded. Second, reads were further filtered by the criterion that 70% of the bases in one read must have high quality scores (≥20). Third, only paired-end reads were used for further assembly. De novo transcript assembly was conducted using Trinity software^51^, which consisted of three successive software modules: Inchworm, Chrysalis, and Butterfly.

The unigene sequences of the four tea plant cultivars were searched using BLASTX against the Nt, Nr, Pfam, Swiss-Prot, GO, COG, and KEGG databases (E-value ≤ 1E-5) to retrieve protein functional annotatio ns based on sequence similarity.

### Gene expression quantification and differential expression analysis

To quantify transcript abundance, the sequenced pair-end reads were mapped onto the assembled transcriptome, and the read count for each gene was obtained from the mapping results. Mapped reads were used for quantification by RSEM software^52^. Gene or isoform abundance was represented by the fragment per kilobase of transcript per million fragment mapped (FPKM) value, and those transcripts with FPKM values equal to or larger than 0.3 were defined as expressed. Prior to differential gene expression analysis, for each sequenced library, the read counts were adjusted by the edgeR programme package through one scaling normalized factor. Differential expression analysis of two treatments was performed using the DEGseq R package^53^. Three independent biological replicates for each treatment were analysed. The P value was adjusted using a q value (Storey *et al*. 2003). Q value < 0.005 was set as the threshold for significantly different expression.

### Validation of RNA-seq analysis by qPCR

qPCR assays were performed to confirm the RNA-seq results. *C. japonica* leaves acclimated at 4 °C for 0, 2, 8, 24, 72 and 168 h were sampled and used for RNA extracting. 5 μg of total RNA was treated with 10 U of DNase I (Thermo Scientific, USA) to remove residual DNA and then used for reverse transcription with TransScript First-Strand cDNA Synthesis Super Mix (TransGen). qRT-PCR was performed as described previously^54^. Three independent biological replicates for each sample and three technical replicates for each biological replicate were analyzed. The *C. Japonica* 18S RNA gene was used as the internal control. All of the primers that were used are listed in Supplementary Table 8.

### Fatty acid analysis

*C. japonica* leaves acclimated at 4 °C for 0, 2, 8, 24, 72 and 168 h were sampled and used for lipid extraction and profiling. The fatty acid extraction was performed following a described protocol^55^. Briefly, samples of ~70 mg fresh weight were ground and then heated at 90 °C in 2.5% (v/v) H_2_SO_4_ in methanol for 90 min in screw-capped tubes. After the addition of 500 μl of hexane containing 0.01% butylated hydroxytoluene, fatty acids were extracted into the organic phase by shaking and the tubes were centrifuged at low speed. The samples were then analyzed using an Agilent 7890 series gas chromatograph. Samples (5 μl) of the organic phase were separated by gas chromatography on a 30 m × 0.25 mm, 0.25 μm film Agilent J&W GC column (USA) and quantified using a flame ionization detector (FID). The gas chromatograph was programmed for an initial temperature of 180 °C for 1 min followed by an increase of 10 °C/min to 220 °C; this final temperature was maintained for a further 4 min. Glyceryl triheptadecanoate (Sigma-Aldrich: T2151) was used as the internal standard.

### JA determination

Sample preparation and JA content quantitation were performed as described previously^56^. Leaves of the *C. japonica* acclimated at 4 °C for 0, 2, 8, 24, 72 and 168 h were sampled, and samples of ~100 mg fresh weight were frozen with liquid nitrogen and stored at −80 °C before analysis. The tissues were ground in 800 μl of pre-cooled extraction buffer (methanol:ddH_2_O:acetic acid, 80:19:1, v/v/v) supplied with 9,10-dihydro-JA (DHJA; Olchemin: 014 5321) as internal standard with ceramic beads (3 mm) using a Tissue Lyser (JXFSTPRP-192; China) for 90 s; the mixture was incubated at 4 °C for 12 h with shaking in the dark. After centrifugation (10,000× g) at 4 °C for 10 min, the supernatant was transferred to a fresh tube; the pellet was mixed with another 400 μl of pre-cooled extraction buffer without internal standard, shaken for 2 h at 4 °C in the dark, and centrifuged. After drying the combined supernatants with nitrogen gas, the pellet was resuspended in 300 μl of pre-cooled 10% methanol and filtered using a syringe-facilitated 13 mm diameter nylon filter with a pore size 0.22 μm. The prepared samples were detected using a 4000Q-TRAP HPLC-MS system (AB SCIEX) and the parameter settings were followed as described previously^57^. Pure JA (Sigma-Aldrich: 14631) was used as external standard to generate the standard curve to quantify the JA content.

### Statistics

Each graphical plot represents the results of multiple independent experiments (n ≥ 3), and the values are means ± SE. Statistical significance of physiological parameters (MDA and relative conductivity) was determined using two-tailed unpaired Student’s *t*-tests, and *P* values pf < 0.05 were considered statistically significant. The fatty acid compositions in different sampling points were compared using a one-way ANOVA and shortest significant range (P < 0.05) post hoc analysis, and values not sharing a common letter were considered statistically significant.

## Additional Information

**How to cite this article**: Li, Q. *et al*. RNA-seq based transcriptomic analysis uncovers α-linolenic acid and jasmonic acid biosynthesis pathways respond to cold acclimation in *Camellia japonica. Sci. Rep.* **6**, 36463; doi: 10.1038/srep36463 (2016).

**Publisher’s note**: Springer Nature remains neutral with regard to jurisdictional claims in published maps and institutional affiliations.

## Supplementary Material

Supplementary Materials

Supplementary Table 5

Supplementary Table 7

## Figures and Tables

**Table 1:** Comparison of fatty acid composition in *C. japonica* leaves during cold acclimation.

		16:0	18:0	18:1	18:2	18:3
‘Jiangxue’	CA0	37.30 ± 1.63^a^	5.08 ± 0.68^a^	14.70 ± 0.70^a^	8.46 ± 0.34^a^	33.96 ± 1.46^a^
	CA2	29.72 ± 1.33^b^	4.11 ± 0.40^ab^	10.45 ± 0.71^b^	7.92 ± 0.13^a^	47.27 ± 1.24^ab^
	CA8	29.16 ± 1.06^b^	3.36 ± 0.30^bc^	9.5 ± 0.41^b^	7.16 ± 0.22^a^	50.71 ± 1.90^ab^
	CA24	27.12 ± 0.81^b^	2.63 ± 0.13^bc^	10.09 ± 0.67^b^	7.64 ± 0.47^a^	51.69 ± 1.23^ab^
	CA72	25.27 ± 0.73^b^	2.70 ± 0.19^bc^	9.08 ± 0.79^b^	8.63 ± 0.56^a^	53.35 ± 1.31^b^
	CA168	24.67 ± 0.57^b^	2.27 ± 0.09^c^	8.16 ± 0.78^b^	8.28 ± 0.33^a^	56.00 ± 1.24^c^
‘Desire’	CA0	43.10 ± 0.70^a^	16.07 ± 0.13^a^	7.09 ± 0.22^ab^	10.66 ± 0.23^ab^	23.07 ± 0.82^a^
	CA2	42.81 ± 3.49^ab^	16.31 ± 0.73^ab^	6.75 ± 1.63^b^	8.13 ± 1.33^c^	24.99 ± 1.26^a^
	CA8	37.98 ± 0.24^bc^	15.89 ± 0.62^abc^	10.07 ± 0.92^a^	9.02 ± 0.83^bc^	27.04 ± 1.10^a^
	CA24	37.19 ± 1.40^bc^	15.66 ± 0.36^abc^	9.53 ± 0.52^a^	11.34 ± 0.60^ab^	26.27 ± 1.57^a^
	CA72	35.48 ± 0.70^bc^	13.80 ± 0.18^bc^	10.09 ± 0.57^a^	11.51 ± 0.42^ab^	29.13 ± 0.63^a^
	CA168	34.13 ± 0.67^c^	13.72 ± 0.50^c^	9.87 ± 0.30^a^	12.85 ± 0.37^a^	29.43 ± 0.63^a^
‘Nuccio’s Bella Rossa’	CA0	44.72 ± 1.42^a^	15.11 ± 0.89^a^	13.48 ± 0.83^a^	4.32 ± 0.76^a^	22.38 ± 0.50^ab^
	CA2	41.51 ± 0.99^a^	15.66 ± 0.23^a^	15.25 ± 0.25^a^	3.58 ± 0.30^a^	24.01 ± 0.64^b^
	CA8	41.14 ± 0.29^a^	15.24 ± 0.19^a^	14.90 ± 0.24^a^	3.24 ± 0.34^a^	25.48 ± 0.67^b^
	CA24	39.16 ± 0.64^ab^	15.95 ± 0.41^a^	13.20 ± 0.34^a^	3.28 ± 0.31^a^	28.42 ± 1.19^ab^
	CA72	36.25 ± 0.49^ab^	14.98 ± 0.11^a^	14.84 ± 0.48^a^	3.90 ± 0.05^a^	30.03 ± 0.81^a^
	CA168	34.61 ± 1.48^b^	12.64 ± 0.27^a^	16.33 ± 1.18^a^	3.44 ± 1.95^a^	32.97 ± 3.52^ab^

Values are expressed in percentage. Data reported are mean valuse of six independent experiments ± SE. Values not sharing a common letter indicate a significant difference (shortest significant range; P < 0.05).
