# Supplementary material for: RNA-seq based transcriptomic analysis uncovers α-linolenic acid and jasmonic acid biosynthesis pathways respond to cold acclimation in Camellia japonica
Source: Sci Rep. 2016 Nov 7;6:36463. doi: 10.1038/srep36463 (PMC5098223; doi:10.1038/srep36463)
Supplement: Supplementary Materials [file srep36463-s1.pdf]

RNA-seq based transcriptomic analysis uncovers α-linolenic acid and jasmonic acid biosynthesis pathways respond to cold acclimation in *Camellia japonica*

Qingyuan Li[1], Sheng Lei[1, 2], Kebing Du[2], Xufeng Pang[1, 2], Lizhi Li[1, 2], Zhanchang Wang[1], Ming Wei[1], Shao Fu[1], Limin Hu[3], and Lin Xu[1]*

[1] Forestry and Fruit Tree Research Institute, Wuhan Academy of Agricultural Science and Technology, Wuhan 430075, China

[2] College of Horticulture and Forestry Sciences, Huazhong Agricultural University, Wuhan 430070, China

[3] National Key Laboratory of Crop Genetic Improvement, Wuhan 430070, China

*Correspondence: Lin Xu. *e-mail:* xulin@wuhanagri.com

Supplementary information

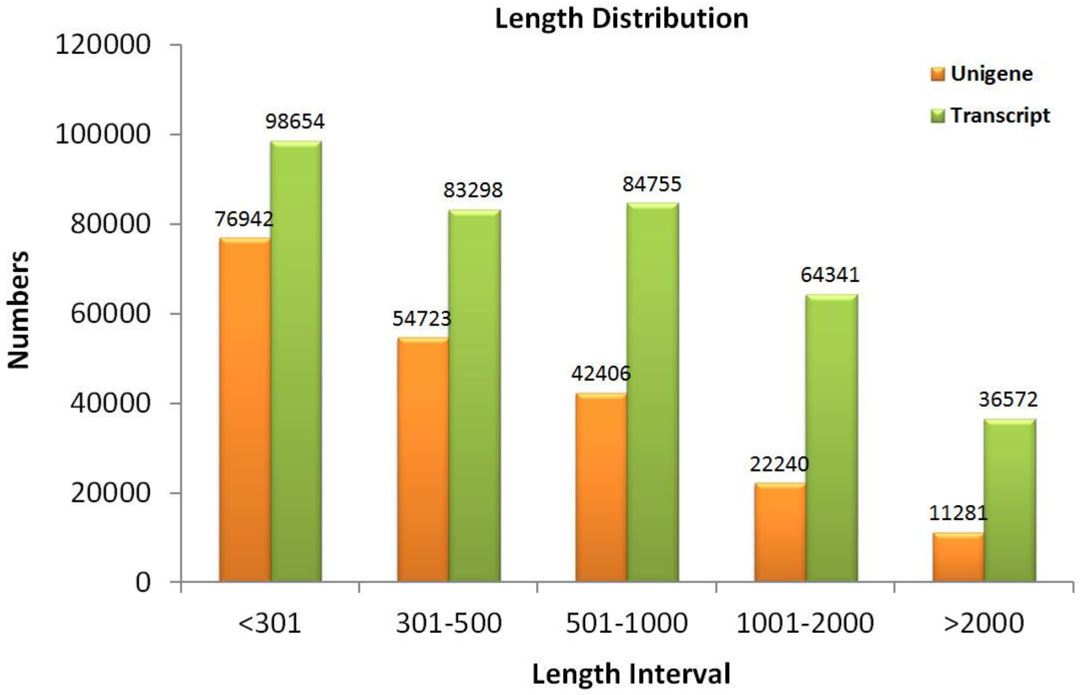

Supplementary Figure 1. Length distribution of the assembled unigenes.

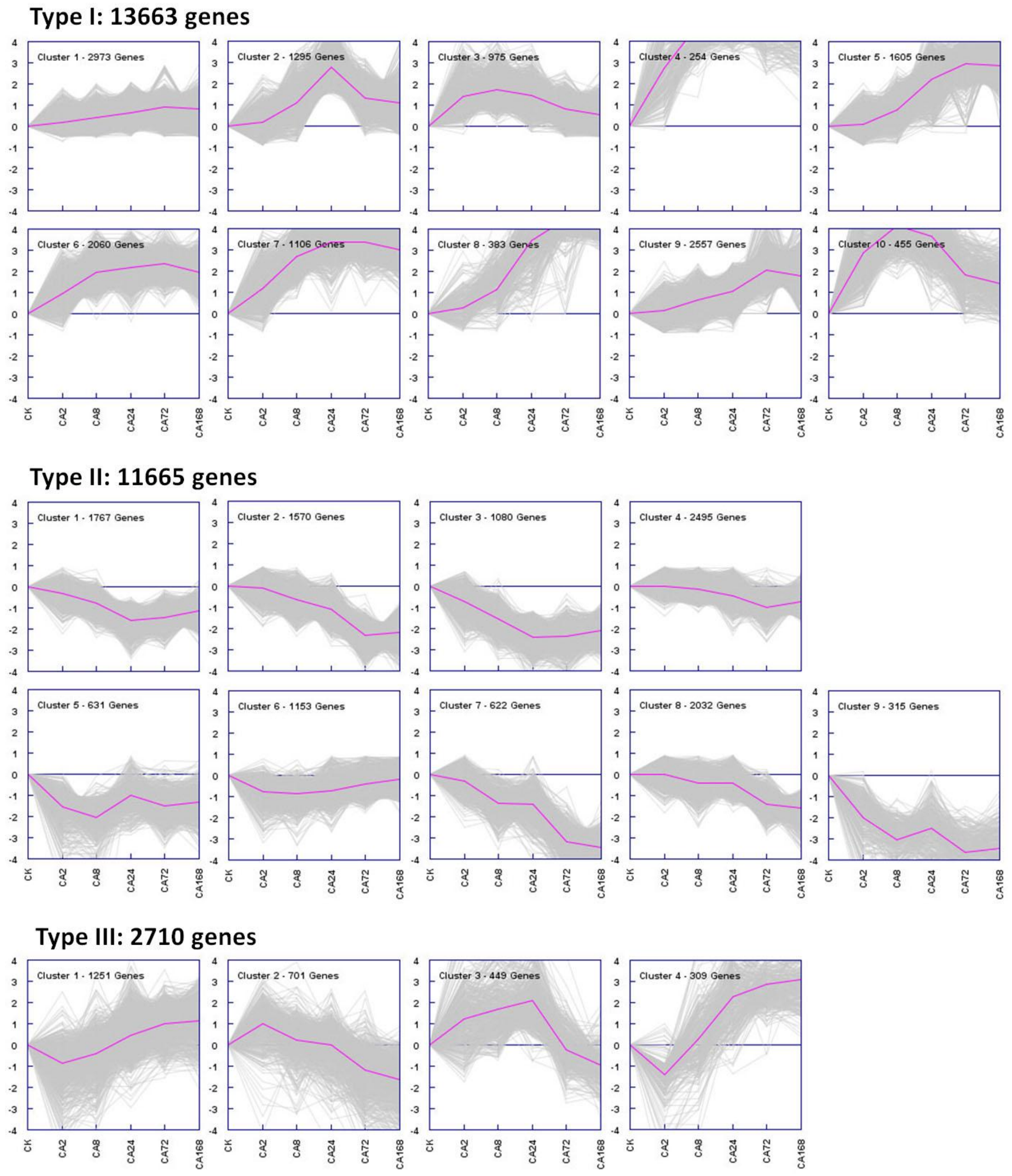

Supplementary Figure 2. Cluster analysis was developed by the K-means method on the gene expression profiles. CK indicates the non-acclimation control, and CA2, CA8, CA24, CA72 and CA168 refer to 2, 8, 24, 72 and 168 h of cold acclimation.

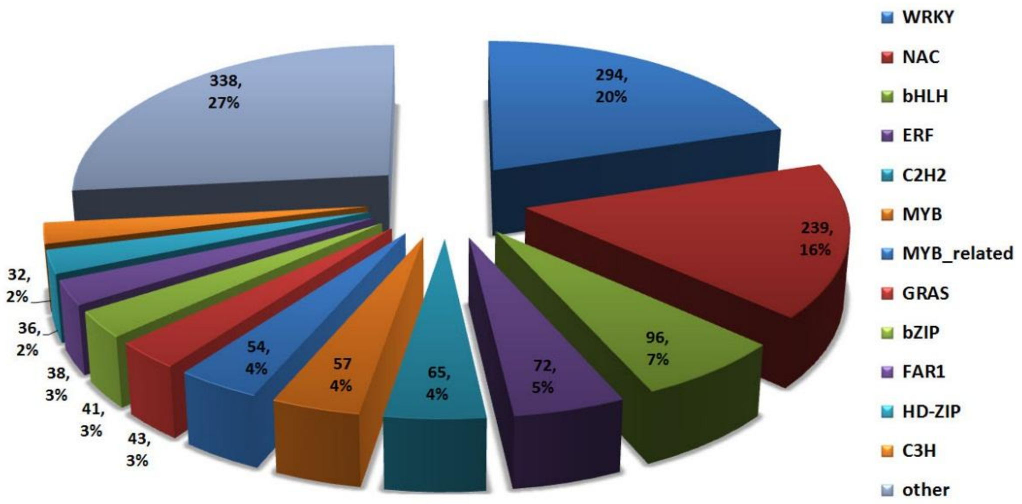

Supplementary Figure 3. Differentially expressed TF genes and classification of TF families during cold acclimation in *C. japonica*. TF genes were classified into TF families by using publicly available Arabidopsis TF databases [Plant Transcription Factor Database (PlnTFDB, http://planttfdb.cbi.pku.edu.cn/index.php)].

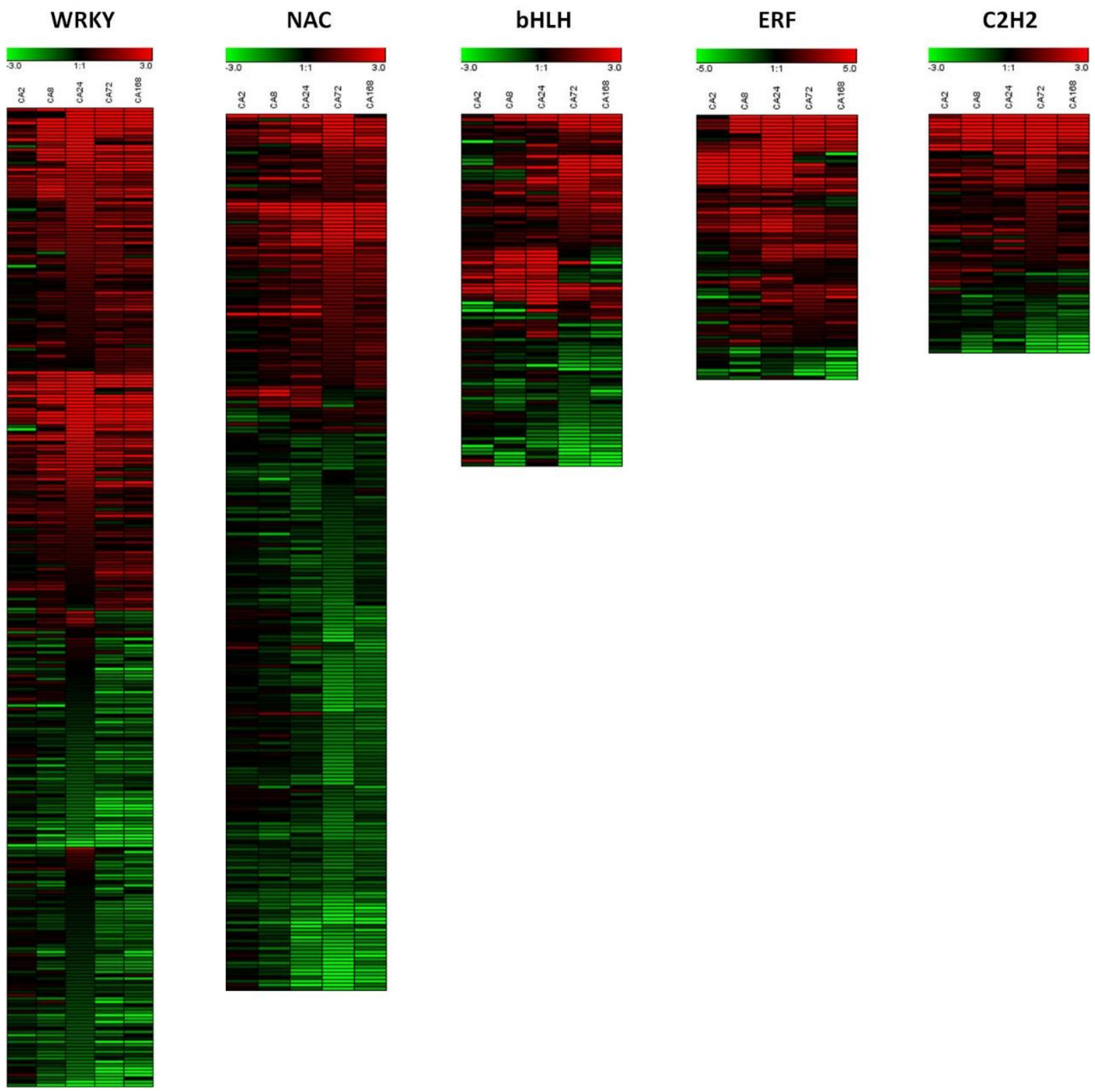

Supplementary Figure 4. Heat maps of the TFs invole in the response to cold acclimation in *C. japonica*.

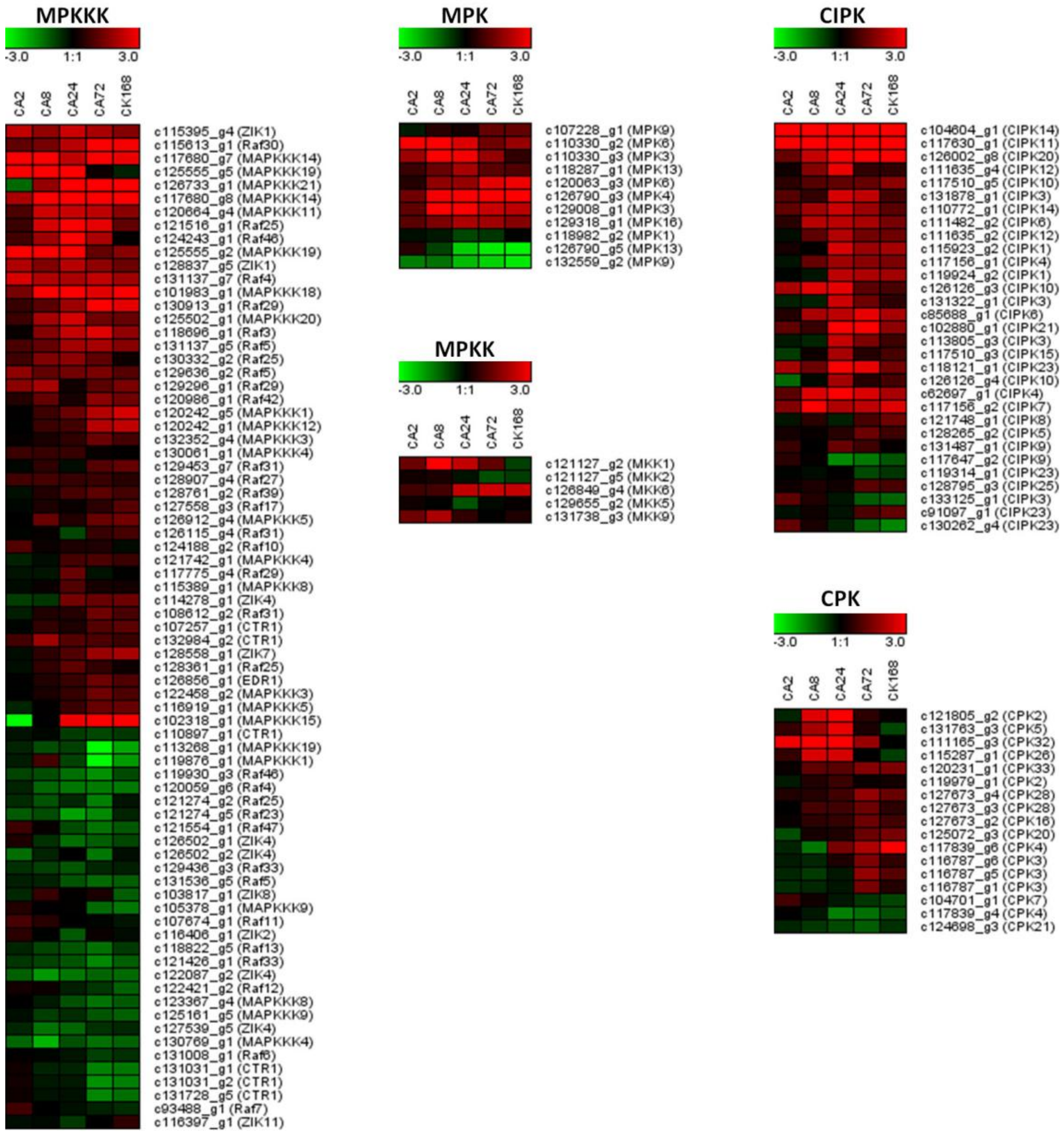

Supplementary Figure 5. Heat maps of the protein kinase invole in the response to cold acclimation in *C. japonica*.

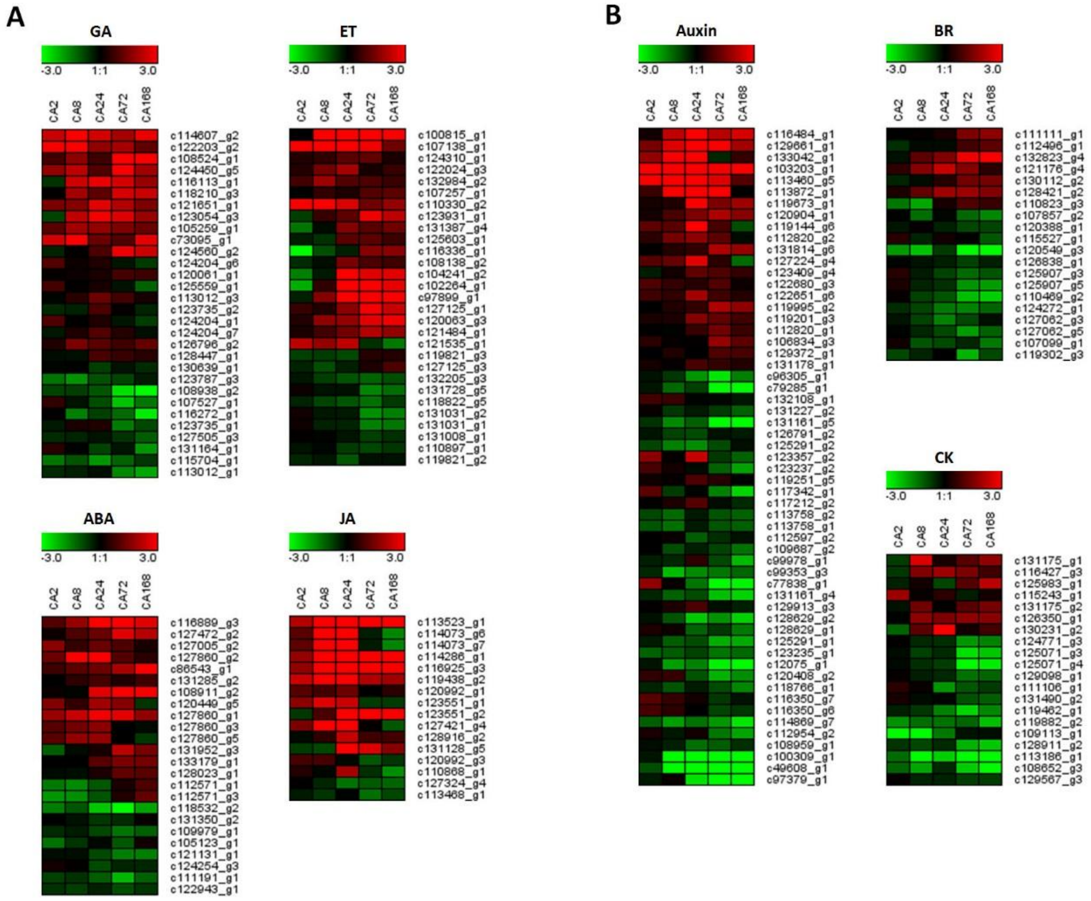

Supplementary Figure 6. Plant hormone signal transduction pathway genes in cold responses in *C. japonica*.

Supplementary Table 1. Detailed climatological data from December 1, 2013 to January 9, 2014.

Year	Month	Date	Average temperature (°C)	Maximum temperature (°C)	Minimum temperature (°C)	Average relative humidity
2013	12	1	6.3	17.2	-0.1	76
2013	12	2	7.6	19.5	1	80
2013	12	3	9.5	18.7	3.2	70
2013	12	4	6.8	16.6	1.2	80
2013	12	5	8.5	16.6	2	73
2013	12	6	5.7	15.7	0.5	78
2013	12	7	5.8	17.2	-0.7	80
2013	12	8	9.2	18.9	2.8	82
2013	12	9	9	13.7	3.1	60
2013	12	10	4.8	12.1	-2.1	67
2013	12	11	4.7	12.8	-0.4	73
2013	12	12	6.8	13.1	0.8	75
2013	12	13	5.1	14.9	-0.8	76
2013	12	14	6.6	13.6	0.5	72
2013	12	15	9.4	12.7	6.1	55
2013	12	16	7.6	10	6.2	77
2013	12	17	6.7	8.3	5.7	78
2013	12	18	3.1	9.5	-1.2	77
2013	12	19	1.2	7.6	-3.1	84
2013	12	20	2.7	9	-1.8	75
2013	12	21	0.2	7.8	-4	77
2013	12	22	-0.4	8.1	-5.5	77
2013	12	23	0.6	10.1	-4.5	74
2013	12	24	1.3	10.9	-4.2	73
2013	12	25	2.7	10.5	-2.9	71
2013	12	26	4	8.7	0.5	52
2013	12	27	-1.9	5.9	-6.8	59
2013	12	28	-1.4	8.7	-6.9	68
2013	12	29	0.5	10.3	-6.3	68
2013	12	30	2.3	13	-4.6	69
2013	12	31	3.7	15.8	-4	76
2014	1	1	4.4	17.9	-2.6	74
2014	1	2	4.4	15.5	-2.5	75
2014	1	3	6	16.2	-1	64
2014	1	4	4.5	16	-1.1	62
2014	1	5	4.1	16.1	-3.5	66
2014	1	6	6.9	10.2	2.9	67
2014	1	7	5.4	6.2	5	93
2014	1	8	4.7	7.8	1.2	82
2014	1	9	1.7	8.8	-2.3	85

Supplementary Table 2. Output quality of RNA-seq data.

Sample	Total raw Reads	Total clean reads	Total clean bases (Gb)	Error(%)	Q20(%)	Q30(%)	GC(%)
CK1	58,712,214	57,304,692	7.16	0.01	97.01	92.61	45.34
CK2	54,782,480	53,739,050	6.72	0.01	97.38	93.44	44.77
CK3	56,293,082	55,159,582	6.89	0.01	97.39	93.41	45.16
L2H1	56,298,426	55,208,138	6.90	0.01	97.45	93.54	45.00
L2H2	57,536,202	56,334,842	7.04	0.01	97.37	93.38	45.29
L2H3	69,672,862	68,363,006	8.55	0.01	97.31	93.22	44.86
L8H1	55,584,540	54,255,022	6.78	0.02	96.83	92.17	44.92
L8H2	61,807,074	60,369,222	7.55	0.01	97.41	93.49	45.17
L8H3	53,608,804	52,141,292	6.52	0.01	97.15	92.97	45.41
L24H1	56,614,676	55,071,386	6.88	0.01	97.24	93.38	44.91
L24H2	54,395,750	52,965,996	6.62	0.01	97.29	93.51	45.06
L24H3	72,436,378	70,155,874	8.77	0.01	97.38	93.70	44.89
L72H1	53,759,510	52,739,760	6.59	0.01	96.86	92.29	45.00
L72H2	56,945,598	55,488,212	6.94	0.01	97.41	93.59	45.14
L72H3	55,365,328	54,088,014	6.76	0.01	97.14	93.04	44.58
L168H1	49,554,492	48,294,164	6.04	0.01	97.32	93.57	44.49
L168H2	48,952,616	47,472,532	5.93	0.01	97.25	93.48	44.83
L168H3	59,073,324	57,569,684	7.20	0.01	97.33	93.60	44.49
Total	1,031,393,356	1,006,720,468	125.84	-	-	-	-
Averge	57,299,631	55,928,915	6.99	0.01	97.25	93.24	44.96

Supplementary Table 3. Summary of length distribution.

	Min length (bp)	Mean length (bp)	Median length (bp)	Max length (bp)	N50 (bp)	N90 (bp)	Total
Transcripts	201	860	507	14487	1415	343	367,620
Unigenes	201	644	374	14487	951	265	207,592

Supplementary Table 4. Summary of unigenes annotation.

	Number of Unigenes	Percentage (%)
Annotated in NR	65150	31.38
Annotated in NT	44101	21.24
Annotated in SwissProt	47011	22.64
Annotated in PFAM	48079	23.16
Annotated in GO	48916	23.56
Annotated in KOG	23514	11.32
Annotated in all Databases	10170	4.89
Annotated in at least one Database	84910	40.9
Total Unigenes	207592	100

Supplementary Table 6. CPK, CIPK, MPK, MPKK and MPKKK genes were identified in the *C. japonica* genome according to the homologous genes in *Arabidopsis* .

Gene ID	*A. thaliana* orthologue	Gene name	BlastP E-value	Gene family
c119979_g1	AT3G10660	CPK2	0	Calcium dependent protein kinase
c121805_g2	AT3G10660	CPK2	2.00E-96	Calcium dependent protein kinase
c116787_g6	AT4G23650	CPK3	1.00E-19	Calcium dependent protein kinase
c116787_g5	AT4G23650	CPK3	3.00E-45	Calcium dependent protein kinase
c116787_g1	AT4G23650	CPK3	7.00E-82	Calcium dependent protein kinase
c117839_g6	AT4G09570	CPK4	2.00E-173	Calcium dependent protein kinase
c117839_g4	AT4G09570	CPK4	5.00E-41	Calcium dependent protein kinase
c131763_g3	AT4G35310	CPK5	1.00E-48	Calcium dependent protein kinase
c104701_g1	AT5G12480	CPK7	8.00E-35	Calcium dependent protein kinase
c127673_g2	AT2G17890	CPK16	9.00E-56	Calcium dependent protein kinase
c125072_g3	AT2G38910	CPK20	0	Calcium dependent protein kinase
c124698_g3	AT4G04720	CPK21	1.00E-157	Calcium dependent protein kinase
c115287_g1	AT4G38230	CPK26	1.00E-15	Calcium dependent protein kinase
c127673_g3	AT5G66210	CPK28	9.00E-82	Calcium dependent protein kinase
c127673_g4	AT5G66210	CPK28	2.00E-51	Calcium dependent protein kinase
c111165_g3	AT3G57530	CPK32	4.00E-12	Calcium dependent protein kinase
c120231_g1	AT1G50700	CPK33	2.00E-89	Calcium dependent protein kinase
c119924_g2	AT3G17510	CIPK1	3.00E-12	CBL-interacting serine-threonine protein kinases
c115923_g2	AT3G17510	CIPK1	1.00E-161	CBL-interacting serine-threonine protein kinases
c133125_g1	AT2G26980	CIPK3	2.00E-128	CBL-interacting serine-threonine protein kinases
c113805_g3	AT2G26980	CIPK3	7.00E-53	CBL-interacting serine-threonine protein kinases
c131322_g1	AT2G26980	CIPK3	8.00E-123	CBL-interacting serine-threonine protein kinases
c131878_g1	AT2G26980	CIPK3	3.00E-98	CBL-interacting serine-threonine protein kinases
c62697_g1	AT4G14580	CIPK4	1.00E-22	CBL-interacting serine-threonine protein kinases
c117156_g1	AT4G14580	CIPK4	7.00E-77	CBL-interacting serine-threonine protein kinases
c128265_g2	AT5G10930	CIPK5	2.00E-82	CBL-interacting serine-threonine protein kinases
c111482_g2	AT4G30960	CIPK6	0	CBL-interacting serine-threonine protein kinases
c85688_g1	AT4G30960	CIPK6	1.00E-33	CBL-interacting serine-threonine protein kinases
c117156_g2	AT3G23000	CIPK7	1.00E-128	CBL-interacting serine-threonine protein kinases
c121748_g1	AT4G24400	CIPK8	1.00E-147	CBL-interacting serine-threonine protein kinases
c131487_g1	AT1G01140	CIPK9	2.00E-136	CBL-interacting serine-threonine protein kinases
c117647_g2	AT1G01140	CIPK9	2.00E-12	CBL-interacting serine-threonine protein kinases
c117510_g5	AT5G58380	CIPK10	8.00E-79	CBL-interacting serine-threonine protein kinases
c126126_g4	AT5G58380	CIPK10	4.00E-148	CBL-interacting serine-threonine protein kinases
c126126_g3	AT5G58380	CIPK10	0	CBL-interacting serine-threonine protein kinases
c117630_g1	AT2G30360	CIPK11	9.00E-164	CBL-interacting serine-threonine protein kinases
c111635_g2	AT4G18700	CIPK12	4.00E-121	CBL-interacting serine-threonine protein kinases
c111635_g4	AT4G18700	CIPK12	6.00E-114	CBL-interacting serine-threonine protein kinases
c110772_g1	AT5G01820	CIPK14	2.00E-176	CBL-interacting serine-threonine protein kinases
c104604_g1	AT5G01820	CIPK14	2.00E-83	CBL-interacting serine-threonine protein kinases
c117510_g3	AT5G01810	CIPK15	1.00E-65	CBL-interacting serine-threonine protein kinases
c126002_g8	AT5G45820	CIPK20	0	CBL-interacting serine-threonine protein kinases
c102880_g1	AT5G57630	CIPK21	4.00E-13	CBL-interacting serine-threonine protein kinases
c119314_g1	AT1G30270	CIPK23	3.00E-23	CBL-interacting serine-threonine protein kinases

c130262_g4	AT1G30270	CIPK23	6.00E-30	CBL-interacting serine-threonine protein kinases
c91097_g1	AT1G30270	CIPK23	8.00E-26	CBL-interacting serine-threonine protein kinases
c118121_g1	AT1G30270	CIPK23	2.00E-26	CBL-interacting serine-threonine protein kinases
c128795_g3	AT5G25110	CIPK25	0	CBL-interacting serine-threonine protein kinases
c118982_g2	AT1G10210	MPK1	0	MAP kinase gene family
c110330_g3	AT3G45640	MPK3	8.00E-22	MAP kinase gene family
c129008_g1	AT3G45640	MPK3	1.00E-145	MAP kinase gene family
c126790_g3	AT4G01370	MPK4	1.00E-20	MAP kinase gene family
c120063_g3	AT2G43790	MPK6	4.00E-20	MAP kinase gene family
c110330_g2	AT2G43790	MPK6	6.00E-17	MAP kinase gene family
c107228_g1	AT3G18040	MPK9	2.00E-14	MAP kinase gene family
c132559_g2	AT3G18040	MPK9	3.00E-35	MAP kinase gene family
c126790_g5	AT1G07880	MPK13	1.00E-24	MAP kinase gene family
c118287_g1	AT1G07880	MPK13	1.00E-107	MAP kinase gene family
c129318_g1	AT5G19010	MPK16	4.00E-110	MAP kinase gene family
c121127_g2	AT4G26070	MKK1	3.00E-13	MAP kinase kinase gene family
c121127_g5	AT4G29810	MKK2	2.00E-148	MAP kinase kinase gene family
c129655_g2	AT3G21220	MKK5	3.00E-75	MAP kinase kinase gene family
c126849_g4	AT5G56580	MKK6	1.00E-141	MAP kinase kinase gene family
c131738_g3	AT1G73500	MKK9	3.00E-122	MAP kinase kinase gene family
c120242_g5	AT1G09000	MAPKKK1	2.00E-12	MAP kinase kinase kinase gene family
c119876_g1	AT1G09000	MAPKKK1	9.00E-87	MAP kinase kinase kinase gene family
c122458_g2	AT1G53570	MAPKKK3	1.00E-77	MAP kinase kinase kinase gene family
c132352_g4	AT1G53570	MAPKKK3	1.00E-85	MAP kinase kinase kinase gene family
c130061_g1	AT1G63700	MAPKKK4	3.00E-105	MAP kinase kinase kinase gene family
c121742_g1	AT1G63700	MAPKKK4	6.00E-79	MAP kinase kinase kinase gene family
c130769_g1	AT1G63700	MAPKKK4	3.00E-39	MAP kinase kinase kinase gene family
c126912_g4	AT5G66850	MAPKKK5	3.00E-53	MAP kinase kinase kinase gene family
c116919_g1	AT5G66850	MAPKKK5	6.00E-176	MAP kinase kinase kinase gene family
c123367_g4	AT4G08500	MAPKKK8	9.00E-16	MAP kinase kinase kinase gene family
c115389_g1	AT4G08500	MAPKKK8	3.00E-35	MAP kinase kinase kinase gene family
c105378_g1	AT4G08480	MAPKKK9	2.00E-21	MAP kinase kinase kinase gene family
c125161_g5	AT4G08480	MAPKKK9	6.00E-13	MAP kinase kinase kinase gene family
c120664_g4	AT4G12020	MAPKKK11	7.00E-39	MAP kinase kinase kinase gene family
c120242_g1	AT3G06030	MAPKKK12	9.00E-165	MAP kinase kinase kinase gene family
c117680_g7	AT2G30040	MAPKKK14	2.00E-115	MAP kinase kinase kinase gene family
c117680_g8	AT2G30040	MAPKKK14	6.00E-124	MAP kinase kinase kinase gene family
c102318_g1	AT5G55090	MAPKKK15	5.00E-106	MAP kinase kinase kinase gene family
c101983_g1	AT1G05100	MAPKKK18	1.00E-108	MAP kinase kinase kinase gene family
c125555_g2	AT5G67080	MAPKKK19	1.00E-83	MAP kinase kinase kinase gene family
c125555_g5	AT5G67080	MAPKKK19	5.00E-115	MAP kinase kinase kinase gene family
c125502_g1	AT3G50310	MAPKKK20	9.00E-74	MAP kinase kinase kinase gene family
c126733_g1	AT4G36950	MAPKKK21	2.00E-66	MAP kinase kinase kinase gene family
c115395_g4	AT3G51630	ZIK1	2.00E-17	MAP kinase kinase kinase gene family
c128837_g5	AT3G51630	ZIK1	6.00E-169	MAP kinase kinase kinase gene family
c116406_g1	AT5G58350	ZIK2	4.00E-39	MAP kinase kinase kinase gene family
c126502_g2	AT3G04910	ZIK4	6.00E-121	MAP kinase kinase kinase gene family
c122087_g2	AT3G04910	ZIK4	4.00E-77	MAP kinase kinase kinase gene family

c114278_g1	AT3G04910	ZIK4	1.00E-65	MAP kinase kinase kinase gene family
c126502_g1	AT3G04910	ZIK4	7.00E-70	MAP kinase kinase kinase gene family
c127539_g5	AT3G04910	ZIK4	1.00E-95	MAP kinase kinase kinase gene family
c128558_g1	AT1G49160	ZIK7	7.00E-16	MAP kinase kinase kinase gene family
c103817_g1	AT5G55560	ZIK8	8.00E-176	MAP kinase kinase kinase gene family
c116397_g1	AT3G48260	ZIK11	0	MAP kinase kinase kinase gene family
c110897_g1	AT5G03730	CTR1	9.00E-65	MAP kinase kinase kinase gene family
c131728_g5	AT5G03730	CTR1	5.00E-23	MAP kinase kinase kinase gene family
c131031_g1	AT5G03730	CTR1	9.00E-62	MAP kinase kinase kinase gene family
c131031_g2	AT5G03730	CTR1	5.00E-65	MAP kinase kinase kinase gene family
c132984_g2	AT5G03730	CTR1	1.00E-148	MAP kinase kinase kinase gene family
c107257_g1	AT5G03730	CTR1	3.00E-26	MAP kinase kinase kinase gene family
c126856_g1	AT1G08720	EDR1	5.00E-31	MAP kinase kinase kinase gene family
c118696_g1	AT5G11850	Raf3	3.00E-19	MAP kinase kinase kinase gene family
c131137_g7	AT1G18160	Raf4	2.00E-11	MAP kinase kinase kinase gene family
c120059_g6	AT1G18160	Raf4	1.00E-26	MAP kinase kinase kinase gene family
c131536_g5	AT1G73660	Raf5	3.00E-29	MAP kinase kinase kinase gene family
c129636_g2	AT1G73660	Raf5	0	MAP kinase kinase kinase gene family
c131137_g5	AT1G73660	Raf5	1.00E-12	MAP kinase kinase kinase gene family
c131008_g1	AT4G24480	Raf6	7.00E-40	MAP kinase kinase kinase gene family
c93488_g1	AT3G06620	Raf7	3.00E-56	MAP kinase kinase kinase gene family
c124188_g2	AT5G49470	Raf10	5.00E-15	MAP kinase kinase kinase gene family
c107674_g1	AT1G67890	Raf11	2.00E-21	MAP kinase kinase kinase gene family
c122421_g2	AT4G23050	Raf12	6.00E-88	MAP kinase kinase kinase gene family
c118822_g5	AT2G31010	Raf13	2.00E-103	MAP kinase kinase kinase gene family
c127558_g3	AT1G14000	Raf17	1.00E-144	MAP kinase kinase kinase gene family
c121274_g5	AT2G31800	Raf23	2.00E-37	MAP kinase kinase kinase gene family
c121516_g1	AT2G43850	Raf25	2.00E-13	MAP kinase kinase kinase gene family
c121274_g2	AT2G43850	Raf25	6.00E-33	MAP kinase kinase kinase gene family
c130332_g2	AT2G43850	Raf25	7.00E-21	MAP kinase kinase kinase gene family
c128361_g1	AT2G43850	Raf25	6.00E-53	MAP kinase kinase kinase gene family
c128907_g4	AT4G18950	Raf27	0	MAP kinase kinase kinase gene family
c130913_g1	AT4G35780	Raf29	6.00E-24	MAP kinase kinase kinase gene family
c129296_g1	AT4G35780	Raf29	2.00E-47	MAP kinase kinase kinase gene family
c117775_g4	AT4G35780	Raf29	4.00E-14	MAP kinase kinase kinase gene family
c115613_g1	AT4G38470	Raf30	9.00E-78	MAP kinase kinase kinase gene family
c126115_g4	AT5G01850	Raf31	1.00E-68	MAP kinase kinase kinase gene family
c108612_g2	AT5G01850	Raf31	4.00E-17	MAP kinase kinase kinase gene family
c129453_g7	AT5G01850	Raf31	7.00E-23	MAP kinase kinase kinase gene family
c121426_g1	AT5G50000	Raf33	1.00E-105	MAP kinase kinase kinase gene family
c129436_g3	AT5G50000	Raf33	8.00E-107	MAP kinase kinase kinase gene family
c128761_g2	AT3G22750	Raf39	0	MAP kinase kinase kinase gene family
c120986_g1	AT3G46920	Raf42	3.00E-30	MAP kinase kinase kinase gene family
c119930_g3	AT3G59830	Raf46	2.00E-67	MAP kinase kinase kinase gene family
c124243_g1	AT3G59830	Raf46	5.00E-69	MAP kinase kinase kinase gene family
c121554_g1	AT3G58760	Raf47	2.00E-97	MAP kinase kinase kinase gene family

Supplementary Table 8. List of primers used in qPCR assays.

Primer	Sequence (5' to 3')	Gene ID
LQP2025	CCCTTCATTTCCCATCCGA	c124803_g2
LQP2026	CCAGTGAGGAGAGGACCATTGT	c124803_g2
LQP2029	TCATAATAGACGCCACGACTCA	c121153_g2
LQP2030	TTACAACAGCAATCACCCCG	c121153_g2
LQP2031	GGCGATGAAAGCCGACGA	c114675_g1
LQP2032	TCCGATTCCGAGACCACCG	c114675_g1
LQP2033	GCCAACTGCTTGATGCTCCT	c128585_g1
LQP2034	CTCGTGGGTCTTCCCCTTTA	c128585_g1
LQP2035	CGCCGCAGCAACAACC	c115655_g1
LQP2036	ATCGGGAACTGGGAATAGAGG	c115655_g1
LQP2037	AGGGTTGGGATGTTTCTTGG	c121292_g2
LQP2038	CTCTGTCTCGTCCCCGTCTC	c121292_g2
LQP2039	AGCAGAAGAAGTGAAGAGGCATTA	c120062_g5
LQP2040	TCCTGTAGTTTGGGTAGGGCAC	c120062_g5
LQP2041	GCAGACGAGTTGAGCAAAGAT	c127146_g10
LQP2042	CATTGGCAAGTGGGTCATACA	c127146_g10
LQP2045	CTCTTGGACCATTGACAACTGATA	c113712_g2
LQP2046	AAACCTTGCCTTGCTTAGTGC	c113712_g2
LQP2049	GCTGCTCATAAGCCCTCCTG	c131064_g2
LQP2050	GATGCTGTTGGCACTCTTGTTC	c131064_g2
LQP2051	TCAATCAGCACATAAATCTTACTCC	c124648_g2
LQP2052	CCCCATCCTGCTAAGGTCAT	c124648_g2
LQP2053	TGGTGGGTAGATGTTTGTGGG	c115468_g1
LQP2054	CATTTTCGCAGGCAGGGA	c115468_g1
LQP2055	TCCTCTGCTGCCTCCTTCG	c119977_g1
LQP2056	TTTTCCTTCTCCACAAACCCC	c119977_g1
LQP2057	TGGAAGCGAAGTGAGGGAAG	c116938_g4
LQP2058	GGTTTTACCATATCATTGGGAGC	c116938_g4
LQP2059	GGCTTTGCCTCGGGTTCA	c122550_g5
LQP2060	TGGTGCTGTGAAGCGGGT	c122550_g5
LQP2063	CCGAGGCAACAAACTGGCT	c122240_g2
LQP2064	CGAGACCGTATGCGAATAGGAG	c122240_g2
LQP2065	AACAACTGGTGGGTGGCATT	c115110_g2
LQP2066	CCGTGGCGAGCAGAAAAC	c115110_g2
LQP2067	TAGACTCCGAGCCGCTGTGTA	c122860_g4
LQP2068	GCCAGCCCACTTGATGTATGA	c122860_g4
LQP2069	GTTGTCTTAGGCTCAAGGCTCAC	c117148_g2
LQP2070	GGACTCCTCATCTTCACTACCGT	c117148_g2
LQP2071	ATGCCGTTGATGAAATCCGTA	c119308_g2
LQP2072	CAAAGGCTGCGTCGTGAGA	c119308_g2
LQP2073	AACCCTTCCCCTTCAACCC	c130291_g3
LQP2074	GAGCCCTTAATTTCACGACACTG	c130291_g3
LQP2075	GCAGGGGAAATGGAGTGGAC	c126366_g3
LQP2076	CAATGAAGCCGTGCTAAAGGA	c126366_g3
LQP2077	GGCTTCCACGGTCTCGCT	c122188_g1

LQP2078	TCCAACCCCCTTTTCTCCC	c122188_g1
LQP2079	CCGTCGCAATAGGTCCAGGC	c132434_g1
LQP2080	GAGGCGGAGGAGGGAGAATCA	c132434_g1
LQP2081	AGGAATGGAGTTATCTGCGAGG	c129820_g1
LQP2082	TGGTAGTGTGGGATTTGAGGG	c129820_g1
LQP2083	GCCACCATTGACACGAACGA	c126650_g5
LQP2084	CGGGAATCACCGCTAACTGC	c126650_g5
LQP2087	CGCTGACAACATCTCGTCCATT	c117770_g1
LQP2088	CGACACGGCGACTCTGCTT	c117770_g1
LQP2089	AGCCACTATTCCCAACTTCACTAA	c89308_g1
LQP2090	AAAAAATACACGAACACCGAAAA	c89308_g1
LQP2091	TTGCCAGCATTGGATGTTTC	c85245_g1
LQP2092	TTGTCATTATTGGAGCGATTTCT	c85245_g1
LQP2093	TGTTTACCACCAAGTACAGGAC	c130874_g2
LQP2094	GGGTTGAAGAGGCTACGAGAGA	c130874_g2
LQP2097	CTAAATCCTCGGACTTATCGGG	c127824_g5
LQP2098	TTACCAGCACAAACACCACCTG	c127824_g5
LQP2099	CCGCTGAGCAACGGATGA	c122860_g2
LQP2100	TGCCAAGGAGCACGGAGAC	c122860_g2
LQP2101	CCGCCGAATCTGACTGCT	c119918_g1
LQP2102	CAAACTCCCCTCTTTCCCACT	c119918_g1
LQP2105	CTTGGCAGGGGAGAAAGATG	c114797_g1
LQP2106	AGCAACAGGCAACTGAGCG	c114797_g1
LQP2107	GTGAATGGGTTTCTTGTGCTGA	c128426_g1
LQP2108	GGCTTTATTGCTTTTGTGGCTT	c128426_g1
LQP2043	TCTCAACCATAAACGATGCCGACCAG	Cj18S RNA
LQP2044	TTTCAGCCTTGCGACCATACTCCC	Cj18S RNA
